# A multifunctional Fe_3_O_4_@Au-HPG-Glc theranostic platform for MRI/fluorescence-guided photothermal ablation of osteosarcoma

**DOI:** 10.3389/fbioe.2026.1868939

**Published:** 2026-09-15

**Authors:** Shengren Xiong, Xiaomei Zheng, Zhe Wu, Shenghui Wu, Zhongliang Yao, Weilin Zheng

**Affiliations:** 1 Fuzhou Second General Hospital, Fuzhou, Fujian, China; 2 Department of Orthopaedics, Fujian Medical University Union Hospital, Fujian Medical University, Fuzhou, Fujian, China; 3 Department of Orthopedics Sports Injury Division, Fujian Medical University Union Hospital, Fujian Medical University, Fuzhou, Fujian, China; 4 School of Materials and Packaging Engineering, Fujian Polytechnic Normal University, Fuzhou, Fujian, China

**Keywords:** Fe3O4@Au-HPG-Glc, *in vivo*, Nanotecehnology, osteosarcoma, photothermal therapy

## Abstract

**Background:**

The clinical management of osteosarcoma is challenged by its aggressive growth and deep anatomical location. Photothermal therapy offers a minimally invasive approach through localized thermal ablation, but its effectiveness is constrained by poor tissue penetration and inaccurate targeting of deep tumors without real-time guidance.

**Methods:**

We synthesized flower-like Fe_3_O_4_@Au nanoparticles coated with hyperbranched polyglycerol (HPG) and conjugated with glucose (Glc) (Fe_3_O_4_@Au-HPG-Glc). In an osteosarcoma mouse model, the photothermal efficacy of the nanoprobes was evaluated by comparing two groups: the control group receiving phosphate-buffered saline plus laser irradiation (phosphate-buffered saline (PBS) + Laser) and the treatment group receiving Fe_3_O_4_@Au-HPG-Glc nanoprobes plus laser irradiation (NPs + Laser). Intratumoral temperature was monitored by infrared thermography, with longitudinal tracking of tumor volume and body weight. Major organs were examined by H&E staining. Tumor accumulation was assessed via whole-body fluorescence and T_2_-weighted magnetic resonance imaging (MRI); pharmacokinetics and biodistribution were quantified by inductively coupled plasma mass spectrometry (ICP-MS).

**Results:**

The NPs + Laser regimen produced rapid intratumoral heating during the 0–4 min irradiation window and significantly delayed tumor growth compared with the PBS + Laser control group, with stable body weight and no overt organ lesions on H&E staining. Whole-body fluorescence and T_2_-weighted MRI confirmed tumor-site accumulation of the nanoprobes. ICP-MS-based pharmacokinetic and biodistribution analyses provided quantitative exposure context, including systemic clearance, reticuloendothelial system capture, and measurable tumor uptake. Together, these data facilitated interpretation of the subsequent photothermal response.

**Discussion:**

Flower-like Fe_3_O_4_@Au-HPG-Glc enables image-guided photothermal treatment in an osteosarcoma model, supported by quantitative pharmacokinetic and biodistribution measurements and an acceptable short-course safety profile. This work establishes a clear imaging-exposure-response relationship and provides a strong foundation for further optimization of magnetic-optical combination strategies in precision cancer therapy.

## 1 Introduction

Osteosarcoma is the most common primary malignant bone tumor in adolescents and young adults. It poses significant clinical challenges due to aggressive local invasion and early micrometastasis (Beird et al., 2022; Tian et al., 2023; Wen et al., 2022). Despite multimodal treatment combining chemotherapy with limb-salvage surgery, the prognosis remains guarded. Medical imaging plays an indispensable role throughout disease management, from initial staging and therapeutic response assessment to longitudinal surveillance. However, conventional contrast agents lack molecular specificity (Zhang et al., 2011; Zhang et al., 2021). Moreover, single-modality imaging readouts often fail to capture early therapeutic effects that could inform timely clinical decisions (Huang et al., 2025).

Magnetic-plasmonic nanostructures have emerged as a promising platform for integrating diagnostic and therapeutic capabilities (Lee et al., 2025; Lin et al., 2017). Superparamagnetic iron oxide (Fe_3_O_4_) cores provide T_2_-weighted magnetic resonance imaging (MRI) contrast and enable magnetic responsiveness (Lee et al., 2012; Zhang et al., 2024), while plasmonic gold shells efficiently convert near-infrared light into localized heat for photothermal therapy (PTT) (Liang et al., 2012). To ensure biocompatibility and functionality, hydrophilic polymer brushes such as hyperbranched polyglycerol (HPG) stabilize the nanoparticle interface, reduce nonspecific protein adsorption, and provide versatile handles for ligand conjugation. Exploiting the increased glycolytic demand characteristic of malignant cells, glucose (Glc) motifs can be incorporated to enhance tumor-selective uptake.

A critical limitation in conventional image-guided nanotherapy studies is that while imaging confirms tumor-site accumulation, it often provides insufficient quantitative context regarding systemic exposure and tissue distribution when interpreting therapeutic outcomes (Bortot et al., 2023; Dennahy et al., 2022; Yang et al., 2025). As shown in Figure 1, the present study contributes a critical piece to this effort by designing a flower-like Fe_3_O_4_@Au-HPG-Glc nanoprobe specifically engineered for osteosarcoma, and pairing longitudinal dual-modality imaging with rigorous pharmacokinetic and biodistribution quantification. This integrated approach enables photothermal efficacy to be evaluated alongside comprehensive exposure metrics and organ-level distribution patterns.

**FIGURE 1:**
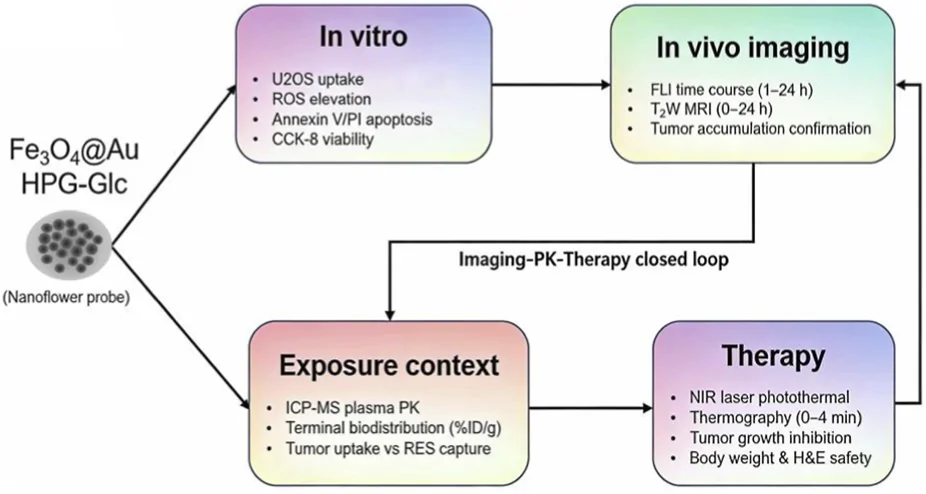
Schematic overview of the Fe_3_O_4_@Au-HPG-Glc platform and the integrated imaging and exposure-informed photothermal evaluation workflow.

Our investigation encompasses three complementary levels: (i) comprehensive physicochemical characterization using transmission electron microscopy (TEM), Fourier-transform infrared spectroscopy (FTIR), ultraviolet-visible spectroscopy (UV-Vis), and room-temperature hysteresis measurements; (ii) *in vitro* mechanistic profiling in U2OS human osteosarcoma cells, establishing the role of HPG–Glc interfacial engineering in mediating cellular responses—including time-dependent uptake, reactive oxygen species (ROS) elevation, and Annexin V/propidium iodide-defined apoptosis; and (iii) *in vivo* imaging and therapeutic assessment employing whole-body fluorescence imaging, T_2_-weighted MRI, pharmacokinetic analysis, biodistribution studies, intratumoral temperature monitoring, tumor growth inhibition measurements, and safety evaluation. Osteosarcoma serves as a clinically relevant, difficult-to-treat model wherein local tumor control and imaging-informed treatment scheduling are particularly critical. All figures and summary statistics were generated through scripted analyses of archived raw data exports to enhance traceability and reproducibility.

Our results demonstrate tumor-site accumulation through dual-modality imaging and show robust intratumoral heating with significant growth inhibition under photothermal conditions, accompanied by stable body weight and absence of overt histopathological changes in major organs. These findings establish Fe_3_O_4_@Au-HPG-Glc as a promising image-guided photothermal therapy platform for osteosarcoma and motivate continued optimization of targeting ligands and dosing paradigms.

## 2 Materials and methods

### 2.1 Materials

Ethylene glycol, diethylene glycol, polyvinylpyrrolidone K30 (PVP), ferric chloride hexahydrate (FeCl_3_·6H_2_O), sodium acetate trihydrate, ethanol, tetrahydrofuran, acetic acid, formaldehyde, hydrogen tetrachloroaurate (III) hydrate (HAuCl_4_·4H_2_O), sodium borate (Na_2_B_4_O_7_·10H_2_O), and other analytical-grade reagents were purchased from Sinopharm Chemical Reagent Co., Ltd. (Shanghai, China) 4-Aminophenyl β-d-glucopyranoside (Glc), glutathione, N-hydroxysulfosuccinimide, 1-ethyl-3-[3-dimethylaminopropyl] carbodiimide hydrochloride, and 3-[4,5-dimethylthiazol-2-yl]-2,5-diphenyltetrazolium bromide (MTT) were obtained from Sigma-Aldrich Chemical Co., Ltd. (St. Louis, MO, United States) U2OS human osteosarcoma cells were obtained from the Chinese Academy of Sciences. Cell culture media and fetal bovine serum were purchased from GIBCO (Shanghai, China). Ultrapure water (UPW) with a resistivity of 18.2 MΩ cm was used throughout all experiments.

### 2.2 Synthesis of flower-like Fe_3_O_4_@Au-HPG-Glc nanoprobes

#### 2.2.1 Synthesis of flower-like Fe3O4 nanoparticles

Flower-like Fe_3_O_4_ nanoparticles were synthesized via a solvothermal method. Briefly, FeCl_3_·6H_2_O (8 mmol) was dissolved in a mixture of ethylene glycol and diethylene glycol (volume ratio 3:17) under ultrasonication at room temperature. After 30 min, PVP (8 g) was added under a nitrogen atmosphere, and the mixture was heated to 125 °C for 1 h. Subsequently, sodium acetate (6 g) was added with increased stirring speed. After 30 min, the mixture was transferred to a 100 mL Teflon-lined stainless-steel autoclave and heated at 200 °C for 12 h. The obtained Fe_3_O_4_ nanoparticles were collected by magnetic separation, washed three times with UPW and ethanol, and redispersed in tetrahydrofuran for storage at 4 °C.

#### 2.2.2 Synthesis of flower-like Fe3O4@Au core-shell nanoparticles

Dopamine modification was first performed to functionalize the Fe_3_O_4_ surface. Dopamine (20 mg) was dissolved in 1.5 mL UPW and added to 5 mL of the Fe_3_O_4_ dispersion, followed by sonication for 1 h and overnight shaking. The resulting Fe_3_O_4_, dopamine was magnetically purified and dispersed in 20 mL UPW. Gold seeds were prepared following a previously reported method (Yin et al., 2017; Zhang et al., 2015). HAuCl_4_ (50 μmol) and glutathione (150 μmol) were mixed in 50 mL UPW and reacted for 3 min. Tetrabutylammonium bromide solution (3 mL) was added, and the mixture was reacted for an additional 5 min. The solution was kept in an ice bath for 2 h, and HCl (1 M) was added dropwise to adjust the pH to 3.0, followed by overnight standing. Negatively charged gold seeds were obtained. For the growth solution, HAuCl_4_ (1.5 mL, 25 mM) was mixed with 0.18 M potassium carbonate solution and stirred for 10 min until the solution became colorless. The growth solution was stored at 4 °C. To synthesize Fe_3_O_4_@Au nanoparticles, the negatively charged gold seeds (200 μL, 12 mg/mL) were added to 2 mL of Fe_3_O_4_-dopamine, followed by the addition of 5 mL UPW and shaking for 5 min. Acetic acid solution (0.2 M) was added dropwise to adjust the pH to approximately 4.0, and the mixture was shaken for 12 h. The resulting Fe_3_O_4_@Au seed nanoparticles were magnetically purified and dissolved in 5 mL UPW. Subsequently, 10 mL of growth solution and 6 mL of formaldehyde solution (1:1 in UPW) were added under vigorous shaking for 30 min to form a dense gold shell.

#### 2.2.3 Synthesis of SH-HPG-COOH dendrimers

Thiol-containing hyperbranched polyglycerol (SH-HPG-COOH) was synthesized via ring-opening polymerization. NaH (0.01 mol, 60% in mineral oil) was added to a dioxane solution (10 mL) containing bis(2-hydroxyethyl) disulfide (120 μL) and heated at 95 °C for 15 min. Glycidol (4.2 mL in 10 mL dioxane) was added dropwise under an argon atmosphere and stirred at 95 °C for 12 h. After removal of the organic phase, the precipitate was dissolved in UPW and dialyzed (MWCO 3 kDa) for 24 h. The obtained disulfide-containing HPG (164 mg) was dissolved in 10 mL UPW under argon, and dithiothreitol (400 μL, 0.5 M) was added. The mixture was reacted at 50 °C for 24 h, followed by dialysis and freeze-drying.

#### 2.2.4 Synthesis of Fe_3_O_4_@Au-HPG-Glc nanoprobes

HPG (6 mg) was added to 1 mL of Fe_3_O_4_@Au nanoparticle dispersion (4 mg/mL) and sonicated for 3 h. The resulting Fe_3_O_4_@Au-HPG was activated using N-hydroxysulfosuccinimide (1.5 mg/mL) and 1-ethyl-3-[3-dimethylaminopropyl] carbodiimide hydrochloride in SBB 9 buffer (50 mM boric acid, pH 9) for 30 min. The activated nanoprobes were magnetically separated and reacted with Glc (1 mg) overnight at room temperature on a rotational mixer. The final Fe_3_O_4_@Au-HPG-Glc nanoprobes were purified by magnetic separation, and their concentration was determined by inductively coupled plasma atomic emission spectrometry (ICP-AES).

### 2.3 Physicochemical characterization

Transmission electron microscopy (TEM) and high-resolution TEM (HRTEM) images were obtained using a JEM-2100F instrument (JEOL, Tokyo, Japan) at an accelerating voltage of 200 kV. Ultraviolet–visible (UV-Vis) absorption spectra were recorded on a Varian Cary 50 spectrophotometer (Varian Inc., Palo Alto, CA, United States). Fourier-transform infrared (FTIR) spectra were collected using a Nicolet 6700 spectrometer (Thermo Scientific, Waltham, MA, United States) with KBr pellets. Dynamic light scattering (DLS) and zeta potential measurements were performed on a NICOMP 380 ZLS particle sizer (PSS Nicomp, Santa Barbara, FL, United States). Magnetization curves were measured using a physical property measurement system (EverCool-II, Quantum Design, San Diego, CA, United States) with a 9 T magnet at 300 K. Nuclear magnetic resonance (NMR) analysis was performed on a Bruker Minispec MQ60 NMR analyzer (1.4 T). Optical microscopy images were taken using a NIKON TS100-F inverted microscope (Nikon, Japan). Clinical magnetic resonance imaging (MRI) was conducted on a Siemens Magnetom Trio 3.0 T MRI system (Siemens, Germany).

### 2.4 Cell culture and in vitro assays

U2OS human osteosarcoma cells were cultured in Dulbecco’s Modified Eagle’s Medium (DMEM) supplemented with 10% fetal bovine serum (FBS), 100 U/mL penicillin, and 100 μg/mL streptomycin. Cells were maintained at 37 °C in a humidified atmosphere containing 5% CO_2_. For cellular uptake and intracellular localization, U2OS cells were seeded in confocal culture dishes at a density of 2 × 10^5^ cells per well and incubated overnight. The culture medium was replaced with fresh medium containing Fe_3_O_4_@Au-HPG-Glc nanoprobes (50 μg Fe/mL). After incubation for 1, 5, or 12 h, cells were washed three times with phosphate-buffered saline (PBS), fixed with 4% paraformaldehyde, and stained with DAPI (nuclei, 1 μg/mL) and LysoTracker (lysosomes, 50 nM). Confocal images were acquired using a Leica TCS SP8 microscope (Leica Microsystems, Wetzlar, Germany) with identical laser power, gain, and pinhole settings across all time points. Intracellular ROS levels were measured using a 2′,7′-dichlorofluorescein diacetate (DCFH-DA) probe. U2OS cells were incubated with Fe_3_O_4_@Au-HPG-Glc nanoprobes (50 μg Fe/mL) for 0, 50, and 150 min. After washing with PBS, cells were incubated with DCFH-DA (10 μM) for 30 min at 37 °C. Fluorescence intensity was analyzed by flow cytometry (BD FACSCalibur, BD Biosciences, Franklin Lakes, NJ, United States) using the FITC channel.

Apoptosis was assessed using Annexin V-FITC/propidium iodide (PI) staining. U2OS cells were treated with nanoprobes (50 μg Fe/mL) for 24 h, harvested, and stained with 5 μL of Annexin V-FITC and 10 μL of PI (20 μg/mL) according to the manufacturer’s instructions. The cells were incubated with the staining solution for 15 min at room temperature in the dark. Stained cells were analyzed by flow cytometry within 1 h. Quadrant analysis was performed to distinguish early apoptotic (Annexin V^+^/PI^−^), late apoptotic (Annexin V^+^/PI^+^), and necrotic (Annexin V^−^/PI^+^) populations.

Cell viability was evaluated using the CCK-8 assay. U2OS cells were seeded in 96-well plates at 1 × 10^4^ cells per well and incubated overnight. Cells were treated with varying concentrations (0–200 μg Fe/mL) of Fe_3_O_4_@Au or Fe_3_O_4_@Au-HPG-Glc for 24 h. CCK-8 solution (10 μL) was added to each well and incubated for 2 h. Absorbance at 450 nm was measured using a microplate reader (BioTek, Winooski, VT, United States). Cell viability was expressed as a percentage relative to untreated control cells. For Live/Dead staining, cells were stained with Calcein-AM (2 μM) and propidium iodide (PI, 4 μM) at 37 °C for 30 min in the dark. Fluorescence images were acquired using an inverted fluorescence microscope. For *in vitro* photothermal performance evaluation, Fe_3_O_4_@Au-HPG-Glc solutions at various concentrations (0–200 μg/mL) were irradiated with an 808 nm laser (1.0 W/cm^2^, 4 min), and temperature changes were recorded. PBS and bare Fe_3_O_4_ core were used as controls.

### 2.5 Animal model and in vivo imaging

All animal experiments were approved by the Institutional Animal Care and Use Committee of Fujian Medical University (protocol number IACUC FJMU 2025-Y-0605) and conducted in accordance with relevant guidelines. Female BALB/c nude mice (6–8 weeks, ∼20 g) were obtained from Henan Skobes Biotechnology Co., Ltd. U2OS cells (5 × 10^6^) suspended in 100 μL PBS were subcutaneously injected into the right flank of each mouse. Tumor volume was monitored using calipers and calculated as (length × width^2^)/2. Experiments were initiated when tumors reached approximately 100 mm^3^. For anesthesia during imaging and laser irradiation, mice were inhaled with isoflurane at a concentration of 2%–5% in oxygen. At the experimental endpoint, mice were euthanized by cervical dislocation under isoflurane anesthesia. For whole-body fluorescence imaging, tumor-bearing mice were intravenously injected with Fe_3_O_4_@Au-HPG-Glc nanoprobes (5 mg Fe/kg body weight). Images were acquired at 1, 2, 4, 8, and 24 h post-injection using an IVIS Lumina III imaging system (PerkinElmer, Waltham, MA, United States) with excitation/emission filters matching the Cy7 fluorophore (λ_ex_ = 750 nm, λ_em_ = 770 nm). Tumor and contralateral regions of interest (ROIs) were analyzed after background correction, and radiance was reported as normalized values. For T_2_-weighted MRI, images were acquired on a 3.0 T clinical MRI system (Siemens Magnetom Trio, Siemens Medical Solutions, Erlangen, Germany) with a small animal coil. Imaging parameters were: repetition time (TR) = 2,600 ms, echo time (TE) = 52 ms, field of view = 1 cm^2^ × 1 cm^2^, slice thickness = 2 mm, matrix = 320 × 256. Images were obtained before injection and at 0, 4, 8, 12, 24, and 48 h post-injection. Tumor signal intensities were normalized to adjacent muscle.

### 2.6 Pharmacokinetics, biodistribution, and photothermal therapy

Serial blood samples were collected from the retro-orbital plexus at predetermined time points (0, 0.25, 0.5, 1, 2, 4, 8, 12, 24, and 48 h) post-injection. Plasma was separated by centrifugation and digested with concentrated nitric acid. At the terminal time point (48 h), mice were euthanized, and major organs (heart, liver, spleen, lung, kidney) and tumors were harvested, weighed, and digested. Fe and Au concentrations were quantified using inductively coupled plasma mass spectrometry (ICP-MS, Agilent 7700, Agilent Technologies, Santa Clara, CA, United States). Pharmacokinetic parameters were calculated using noncompartmental analysis (NCA) with the linear-up/log-down trapezoidal rule.

For photothermal therapy, tumor-bearing mice were randomly divided into two groups (n = 5 per group): (1) PBS + laser (control) and (2) Fe_3_O_4_@Au-HPG-Glc + laser (treatment). For the treatment group, nanoprobes (5 mg Fe/kg body weight) were intravenously injected 24 h prior to laser irradiation. For NIR irradiation, an 808 nm laser (1.0 W/cm^2^, 4 min) was applied to the tumor region. Intratumoral temperature was monitored in real time using an infrared thermal camera (FLIR Systems, Wilsonville, OR, United States). Tumor volumes and body weights were recorded every 2 days for 14 days. Tumor growth inhibition (TGI) was calculated as TGI (%) = 100 × (1 − Δ*V*_treated/Δ*V*_control).

### 2.7 Histological and serum biochemical analysis

At the endpoint, mice were euthanized, and major organs (heart, liver, spleen, lung, kidney) and tumors were harvested, fixed in 4% paraformaldehyde, embedded in paraffin, sectioned at 5 μm thickness, and stained with hematoxylin and eosin (H&E). Histological sections were examined under a light microscope (Nikon Eclipse Ti-S, Nikon, Tokyo, Japan) for pathological evaluation.

For serum biochemical analysis, blood samples were collected from mice immediately after the experimental endpoint and allowed to stand at room temperature until complete coagulation. Serum was then separated by centrifugation at 4 °C and stored for further analysis. Prior to detection, commercial assay kits for alanine aminotransferase (ALT), aspartate aminotransferase (AST), blood urea nitrogen (BUN), and creatinine (Cr) were equilibrated to room temperature along with the serum samples. Working solutions, standards, and quality control samples were prepared according to the manufacturers’ instructions. Serum ALT, AST, BUN, and Cr levels were measured using commercial assay kits according to the manufacturers’ protocols, and concentrations were calculated from the corresponding standard curves.

### 2.8 Statistical analysis

All data are presented as mean ± Standard Deviation (SD). Statistical analyses were performed using SPSS 18.0 (IBM Corp., Armonk, NY, United States). Comparisons between two groups were conducted using two-tailed Student’s t-test. For time-course or multi-group comparisons, one-way or repeated-measures analysis of variance (ANOVA) was used, followed by Dunnett’s *post hoc* or Tukey’s *post hoc* test for multiple comparisons. A p-value <0.05 was considered statistically significant.

## 3 Results and discussion

### 3.1 Characterization of flower-like Fe_3_O_4_@Au-HPG-Glc nanoprobes

The morphology and structure of the Fe_3_O_4_@Au-HPG-Glc nanoprobes were characterized by TEM. As shown in Figure 2A, the synthesized nanoprobes exhibited a well-defined flower-like morphology with a distinct core-shell architecture. The inner core, appearing as a dark contrast region, corresponds to the Fe_3_O_4_@Au heterostructure, while the surrounding lighter shell is attributed to the HPG polymer layer and conjugated Glc molecules, confirming the successful construction of the organic-inorganic hybrid nanoprobe. Energy-dispersive X-ray spectroscopy (TEM-EDS) elemental mapping (Figure 2B) further revealed that Fe, O, and Au signals were co-localized within the same nanoparticle regions, confirming that the Fe_3_O_4_ and Au components are present within the same nanostructure rather than as a physical mixture of individual particles. The successful surface modifications were confirmed by UV-Vis and FTIR spectroscopy. The UV-Vis absorption spectra (Figure 2C) showed that, in addition to the characteristic absorption of the nanoprobes, the Fe_3_O_4_@Au-HPG-Glc sample exhibited a distinct absorption peak corresponding to the glucose (Glc) moiety (the peak in the UV-vis absorption curve approximately 280 nm), confirming the successful conjugation of Glc onto the nanoprobe surface. FTIR spectra (Supplementary Figure S1) further validated the successful functionalization of the nanoprobe surface. Compared with Fe_3_O_4_@Au and Fe_3_O_4_@Au-HPG, the spectrum of Fe_3_O_4_@Au-HPG-Glc exhibited several new absorption features indicative of Glc conjugation. Specifically, peaks at ∼1,510 cm^−1^ were assigned to the stretching vibrations of C=C bonds in the aromatic ring and C-OH bonds of glucose (Lim et al., 2008; Zheng et al., 2025), respectively. The peak at 1,040 cm^−1^ corresponded to the symmetrical stretching vibration of C-O-C bonds, while the band around 1,230 cm^−1^ was attributed to the stretching vibration of C-N in secondary amides (Cheng et al., 2006), confirming the formation of covalent amide bonds between the carboxyl groups of HPG and the amine group of Glc. Collectively, these spectral features confirm the presence of both HPG and Glc on the nanoparticle surface.

**FIGURE 2:**
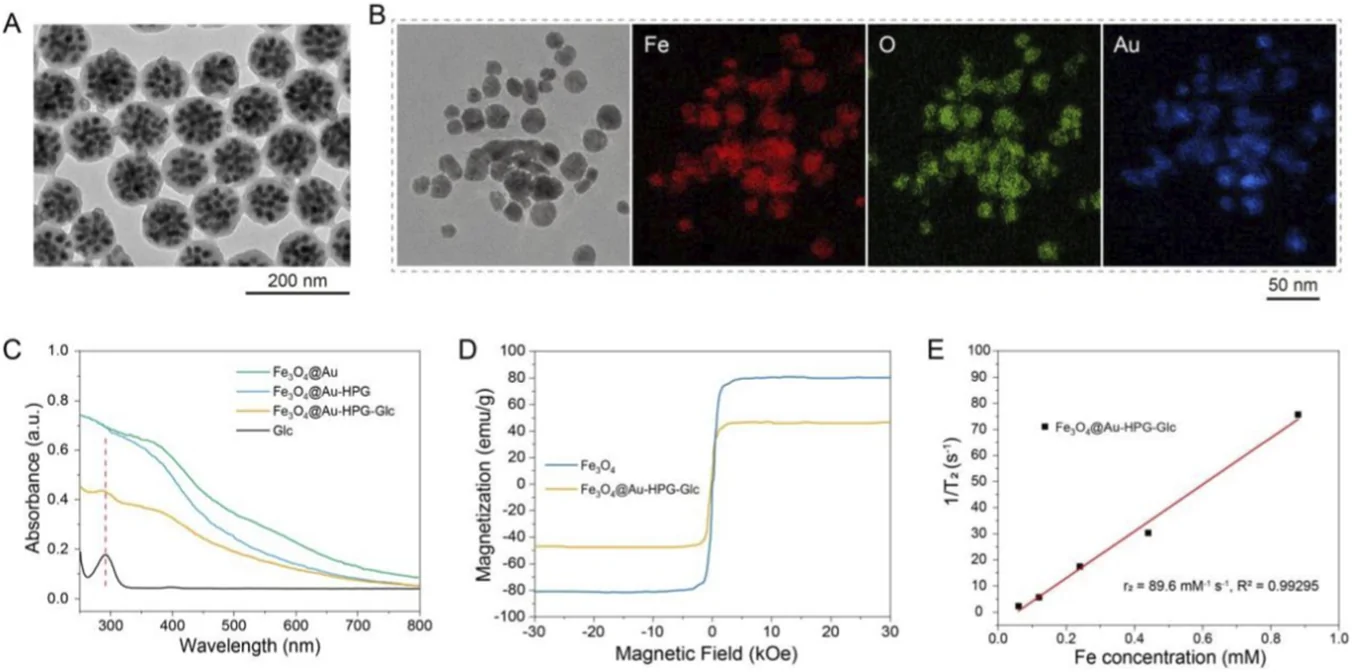
**(A)** TEM images of Fe_3_O_4_@Au-HPG-Glc; **(B)** TEM-EDS elemental mapping showing co-localization of Fe, O, and Au signals in Fe_3_O_4_@Au-HPG-Glc nanoprobes; **(C)** UV–vis absorption of Fe_3_O_4_@Au, Fe_3_O_4_@Au-HPG, Fe_3_O_4_@Au-HPG-Glc, and pure glucose (Glc); **(D)** Magnetization loops of the Fe_3_O_4_ and Fe_3_O_4_@Au-PG-Glc nanoprobes, measured at 300 K. **(E)** Linear fitting of 1/T_2_ for Fe_3_O_4_@Au-HPG-Glc with Fe concentrations of 0.06, 0.12, 0.24, 0.44, and 0.88 mM.

The magnetic properties, crucial for MRI contrast, were evaluated by magnetization loops (Figure 2D). The saturation magnetization values were approximately 80.9 emu g^−1^ for bare Fe_3_O_4_ and 47.5 emu g^−1^ for Fe_3_O_4_@Au-HPG-Glc nanoprobes. The observed reduction in saturation magnetization is expected after coating with non-magnetic organic layers, which dilute the magnetic core content (Yin, et al., 2017). Nevertheless, the nanoprobes retained superparamagnetic behavior with sufficient magnetic moment, meeting the requirements for effective T_2_-weighted MRI contrast enhancement. The linear fitting of 1/T_2_ versus Fe concentration (Figure 2E) gave a high r_2_ relaxivity value, confirming its potential as an excellent T_2_-weighted MRI contrast agent.

### 3.2 Cellular uptake, ROS generation, and apoptosis

To evaluate the interaction of the Fe_3_O_4_@Au-HPG-Glc nanoprobes with osteosarcoma cells, a series of *in vitro* assays were performed. As shown in Figures 3A–C, confocal microscopy revealed time-dependent internalization of the nanoprobes into U2OS cells. At 1 h post-incubation (Figure 3A), the fluorescence signal of the Fe_3_O_4_@Au-HPG-Glc nanoprobes was relatively weak and predominantly localized on the cell membrane surface, indicating the initial binding phase. Meanwhile, the LysoTracker-positive signals (red) remained largely distributed at the periphery of the cells, suggesting limited endocytic activity at this early time point. By 5 h, a marked increase in intracellular nanoprobe fluorescence was observed, and the nanoprobes exhibited substantial co-localization with lysosomes (as indicated by the merged yellow signals in Figure 3B), suggesting that the nanoprobes were internalized via the endo-lysosomal pathway. At 12 h (Figure 3C), both the nanoprobe-derived fluorescence and the lysosomal signals gradually diminished, likely due to gradual degradation of the nanoprobes within the acidic lysosomal environment or their escape from the lysosomal compartment. Notably, this time-dependent trafficking pattern, transitioning from membrane binding at 1 h to lysosomal accumulation at 5 h and subsequent clearance or degradation by 12 h, supports the notion that the nanoprobes are actively internalized through endocytosis rather than passive diffusion (Bai et al., 2023). The lysosomal co-localization observed at 5 h is particularly relevant, as this acidic organelle may serve as the primary site for Fe ion release and subsequent Fenton-reaction-mediated ROS generation (Fu et al., 2025; Gao et al., 2019; Pramanik and Das, 2021). This mechanism was further corroborated by the parallel ROS measurements performed at the same time points (Figure 3D).

**FIGURE 3:**
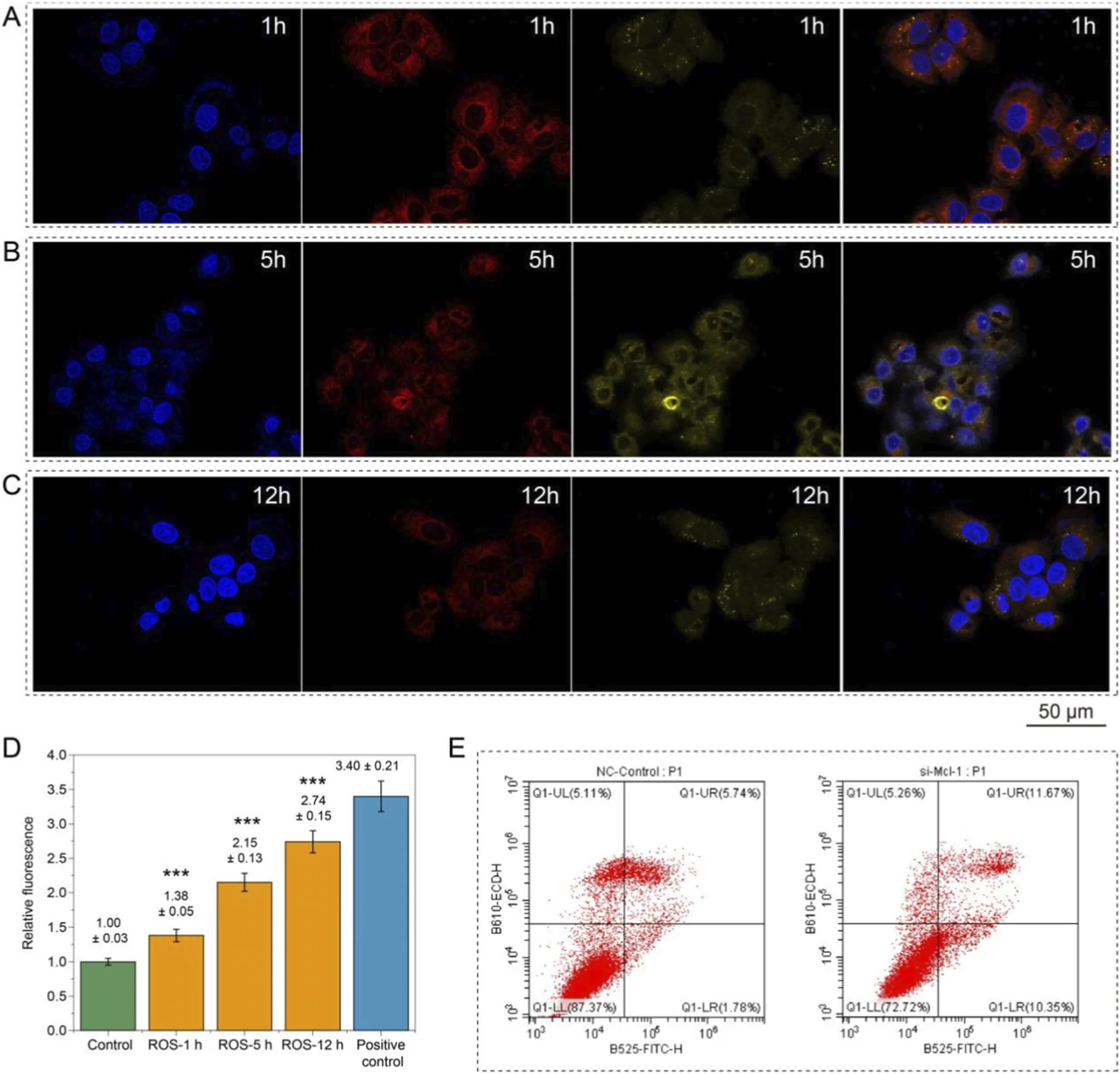
Intracellular trafficking and ROS production of Fe_3_O_4_@Au-HPG-Glc nanoprobes in osteosarcoma cells. Confocal images showing time-dependent internalization and lysosomal co-localization at **(A)** 1 h, **(B)** 5 h, and **(C)** 12 h. The nanoprobes are shown in yellow, lysosomes are labeled with LysoTracker in red, and nuclei are stained with DAPI in blue. The merged signals indicate co-localization of the nanoprobes with lysosomes. **(D)** Relative ROS levels (1/5/12 h) measured by flow cytometry at the same time points. **(E)** Annexin V/PI apoptosis analysis (NC control indicates the untreated cells served as the blank control, si-Mci-1 indicates U2OS cells treated with Fe_3_O_4_@Au-HPG-Glc nanoprobes). Data are mean ± SD (n = 3). Statistical significance was determined by one-way ANOVA followed by Dunnett’s *post hoc* test. ***p < 0.001 vs. control group.

The biological impact of this oxidative stress was further assessed using Annexin V/PI staining (Figure 3E; Supplementary Figure S2). Flow cytometric analysis revealed that nanoprobe treatment induced both early and late apoptosis in U2OS cells, with a marked increase in the percentage of apoptotic cells relative to untreated controls. This apoptotic response aligns with the established role of reactive oxygen species as key activators of intrinsic apoptotic pathways, supporting a mechanistic link between nanoprobe-induced oxidative stress and downstream cell death (Li et al., 2026).

Importantly, CCK-8 assays (Supplementary Figures S3, S4) confirmed that the nanoprobes exhibited acceptable baseline biocompatibility, with cell viability remaining above 80% at the tested concentrations for both Fe_3_O_4_@Au and Fe_3_O_4_@Au-HPG-Glc groups. This indicates that the observed cytotoxicity is not attributable to intrinsic material toxicity but rather arises from the glucose-mediated targeting and the subsequent intracellular events, including enhanced cellular uptake and ROS elevation. Collectively, these *in vitro* findings establish that the Fe_3_O_4_@Au-HPG-Glc nanoprobes achieve targeted cellular effects through a mechanism involving endocytic internalization, oxidative stress, and ultimately apoptosis, while maintaining favorable baseline biocompatibility.

To further evaluate the photothermal anti-tumor efficacy of Fe_3_O_4_@Au-HPG-Glc at the cellular level, we performed CCK-8 viability assay, Live/Dead staining, and Annexin V/PI flow cytometry on U2OS cells after NIR irradiation (808 nm, 1.0 W/cm^2^, 4 min). As shown in Supplementary Figure S5, the Fe_3_O_4_@Au-HPG-Glc plus laser (NPs + Laser) group exhibited a marked reduction in cell viability to approximately 38%, significantly lower than that of the Control group (p < 0.001). Live/Dead staining (Supplementary Figure S6; Supplementary Table S1) further confirmed the extensive cell death induced by photothermal treatment, with a dead cell percentage of 59.4% ± 2.7% in the NPs + Laser group, compared with 5.3% ± 0.6% in the Control group. Annexin V/PI flow cytometry analysis (Supplementary Figure S7; Supplementary Table S2) revealed that the NPs + Laser group induced both early and late apoptosis, with a total apoptosis rate of 51.1% ± 1.6%, whereas the Control, Laser alone, and NPs alone groups showed only minimal apoptosis (3.8%–5.5%). These results confirm that the photothermal effect mediated by Fe_3_O_4_@Au-HPG-Glc efficiently induces U2OS cell death *in vitro*, consistent with the subsequent *in vivo* antitumor efficacy.

### 3.3 *In vivo* imaging, pharmacokinetics, and biodistribution

The tumor-targeting capability of the Fe_3_O_4_@Au-HPG-Glc nanoprobes were first evaluated using *in vivo* imaging modalities. Whole-body fluorescence imaging (Figure 4A) revealed time-dependent accumulation of the nanoprobes at the tumor site, with signal intensity progressively increasing from 1 to 24 h post-injection, reaching its maximum at 24 h. This sustained enrichment suggests efficient tumor homing, likely driven by the combined effect of enhanced permeability and retention (EPR) and the glucose-mediated targeting of metabolically active tumor cells (Cheng et al., 2022; Suzuki et al., 2019).

**FIGURE 4:**
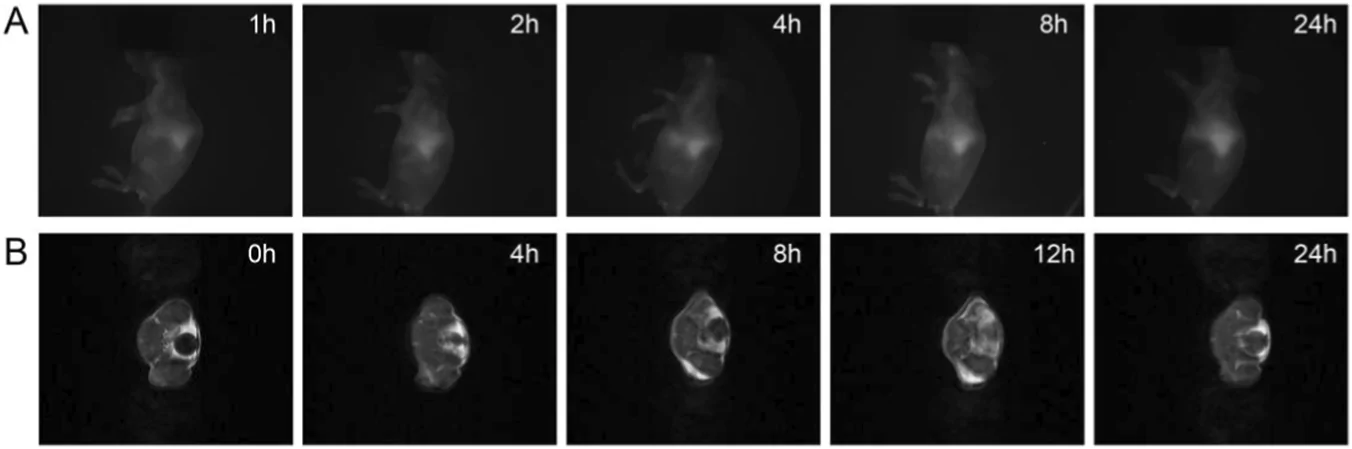
*In vivo* tumor imaging after intravenous injection of Cy7-labeled Fe_3_O_4_@Au-HPG-Glc nanoprobes. **(A)** Whole-body fluorescence imaging at 1, 2, 4, 8, and 24 h post-injection (Cy7, λ_ex_/λ_em_ = 750/770 nm); **(B)** T_2_-weighted MR images from 0 to 24 h, showing progressive hypointensity at the tumor site.

Complementing this observation, T_2_-weighted MRI (Figure 4B and Supplementary Figures S8, S9) demonstrated a progressive hypointensity within the tumor region over 48 h, characteristic of iron-oxide-mediated T_2_ shortening. The gradual darkening correlated temporally with the fluorescence signal increase, providing orthogonal confirmation of sustained nanoprobe retention at the target site. Together, these dual-modality imaging data establish robust tumor accumulation of the nanoprobes, which is a prerequisite for effective photothermal intervention.

To quantitatively contextualize the *in vivo* behavior of the nanoprobes, pharmacokinetic profiling was performed via ICP-MS analysis of plasma Fe concentration (Supplementary Figure S10). The plasma concentration–time curve exhibited a rapid distribution phase immediately following intravenous administration, followed by a slower elimination phase, a profile consistent with the prolonged circulation behavior commonly observed for PEGylated or hyperbranched polymer-coated nanoparticles. This circulation pattern is favorable for passive tumor targeting via the EPR effect.

Endpoint biodistribution analysis (Supplementary Figure S11) revealed that the nanoprobes predominantly accumulated in organs of the reticuloendothelial system, particularly the liver and spleen, which is typical for systemically administered nanoparticles and reflects their clearance by phagocytic cells. Notably, a significant and quantifiable amount of Fe was also detected in the tumor tissue at the terminal time point. Based on ICP-MS quantification of tumor Fe content, the tumor uptake was calculated to be approximately 8.53% ± 0.37% of the injected dose (%ID), corresponding to 7.18 ± 1.45 %ID/g. This measurable tumor uptake provides critical quantitative exposure context, enabling a direct link between the observed therapeutic efficacy and the degree of target-site accumulation. Collectively, these pharmacokinetic and biodistribution data bridge the imaging findings with the subsequent photothermal outcomes, reinforcing the interpretation that tumor suppression is mediated by effective delivery and sustained retention of the nanoprobes at the intended site.

### 3.4 Photothermal therapy and systemic safety

Before evaluating the *in vivo* photothermal efficacy, we first characterized the *in vitro* photothermal properties of the Fe_3_O_4_@Au-HPG-Glc nanoprobes. As shown in Supplementary Figure S12A, Fe_3_O_4_@Au-HPG-Glc solutions at different concentrations (0–200 μg/mL) were irradiated with an 808 nm laser (1.0 W/cm^2^, 4 min), and the temperature rise increased in a concentration-dependent manner, confirming that the photothermal effect correlates with the nanoparticle concentration. Furthermore, compared with PBS and bare Fe_3_O_4_ core under the same irradiation conditions (Supplementary Figure S12B), Fe_3_O_4_@Au-HPG-Glc exhibited significantly higher temperature elevation, confirming that the enhanced photothermal effect is attributable to the Au shell rather than the iron oxide core alone. These *in vitro* data confirm that Fe_3_O_4_@Au-HPG-Glc possesses efficient and concentration-dependent photothermal conversion capability.

Given the confirmed tumor accumulation of the Fe_3_O_4_@Au-HPG-Glc nanoprobes, their photothermal therapeutic efficacy was subsequently evaluated. Infrared thermography (Figure 5A) revealed a rapid temperature rise within the tumor region in the Fe_3_O_4_@Au-HPG-Glc nanoprobe plus laser group during the first 4 min of near-infrared (NIR) irradiation, with temperatures reaching levels sufficient for inducing irreversible tumor ablation. In contrast, the PBS plus laser control group exhibited only a minimal temperature increase under identical irradiation conditions. To further confirm that the enhanced photothermal effect is specifically attributable to the gold shell rather than the iron oxide core, an additional control group (Fe_3_O_4_-core + Laser) was included (Supplementary Figure S13). This group showed only a moderate temperature elevation (42.08 °C ± 0.37 °C) under the same irradiation conditions, markedly lower than that of the Fe_3_O_4_@Au-HPG-Glc + Laser group (53.50 °C ± 0.77 °C). These results indicate that the iron oxide core alone generates insufficient photothermal conversion for effective tumor ablation. The rapid heating capability of Fe_3_O_4_@Au-HPG-Glc nanoprobes is attributable to the plasmonic gold shell, which exhibits strong absorption in the NIR window and enables localized hyperthermia precisely at the target site (Jauffred et al., 2019).

**FIGURE 5:**
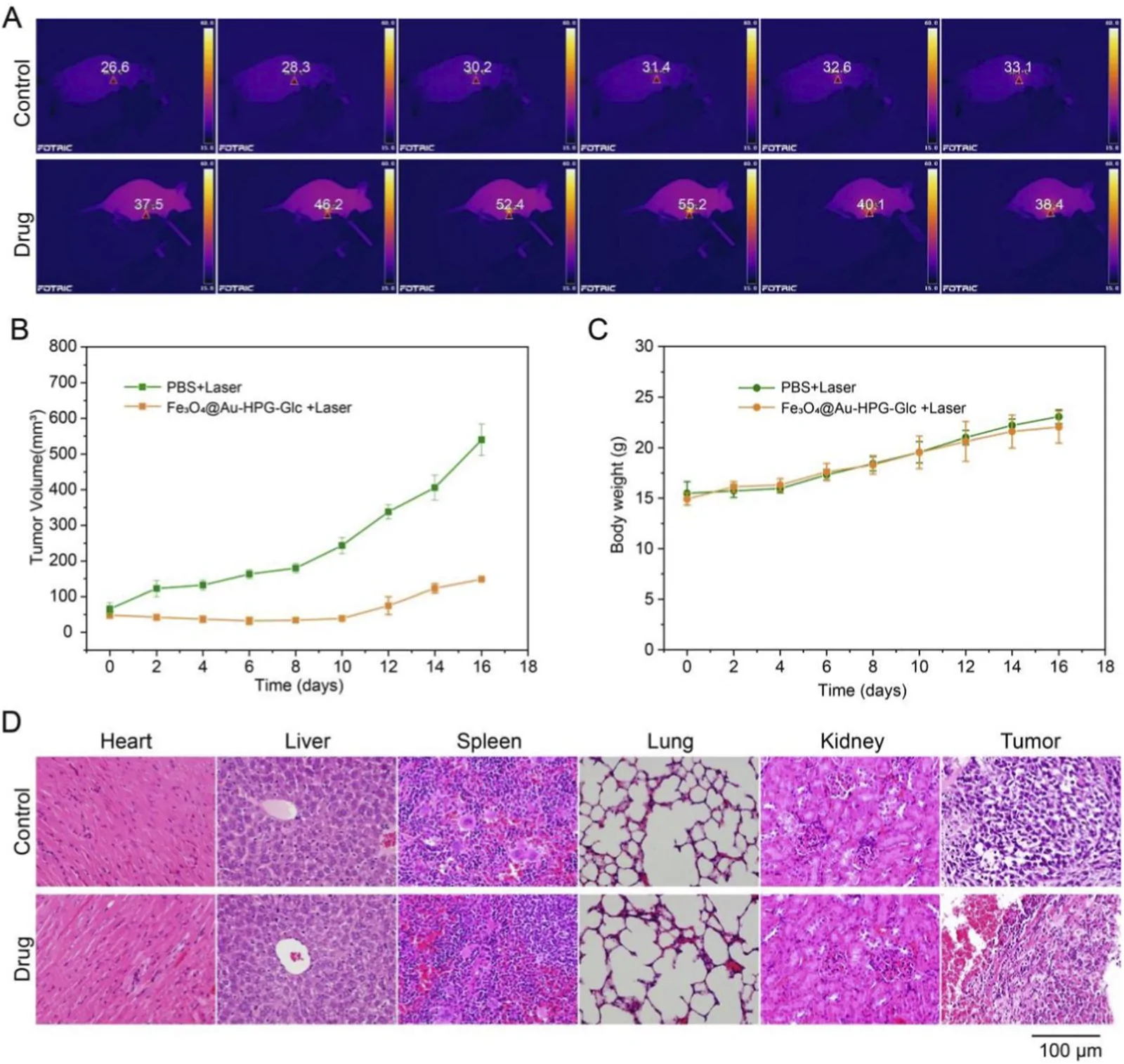
**(A)** Full infrared thermography time series of the tumor region under NIR irradiation. Top row: PBS; bottom row: Fe_3_O_4_@Au–HPG–Glc. Frames at 0, 0.5, 1, 2, 3, and 4 min; **(B)** Growth curves of tumor volume of mice under indicated treatments. Data are presented as mean ± SD (n = 5 mice per group). Statistical significance was determined by repeated-measures two-way ANOVA, with treatment and time as factors. p < 0.001 vs. Control group; **(C)** Changing curves of mice body weight after different treatment. Data are mean ± SD (n = 5 mice per group). No significant difference between groups (p > 0.05); **(D)** H&E staining of major organs of mice treated with PBS (control group) and Fe_3_O_4_@Au-HPG-Glc nanoprobes (Drug group) under NIR irradiation.

Consistent with this differential thermal response, tumor growth curves (Figure 5B) demonstrated that the nanoprobe plus laser group achieved significant tumor growth inhibition compared with the PBS plus laser control group. Notably, laser irradiation alone in the absence of nanoprobes failed to produce substantial antitumor effects, confirming that the observed therapeutic benefit arises specifically from the photothermal conversion mediated by the accumulated nanoprobes within the tumor.

Systemic tolerability of the treatment regimen was assessed through longitudinal monitoring of body weight and post-mortem histopathological analysis. Body weight curves (Figure 5C) remained stable across both groups throughout the observation period, with no significant differences between the nanoprobe-treated and control groups, suggesting the absence of overt systemic toxicity or cachexia typically associated with aggressive therapeutic interventions. Furthermore, H&E staining of major organs (including heart, liver, spleen, lung, and kidney) revealed no obvious pathological abnormalities, inflammatory infiltrates, or structural lesions in the nanoprobe-treated group compared with PBS controls (Figure 5D). These histological findings collectively indicate that the Fe_3_O_4_@Au-HPG-Glc nanoprobes, under the current dosing and irradiation regimen, exhibit a favorable short-term safety profile.

To further evaluate potential subclinical organ damage, particularly given the higher nanoparticle accumulation in the liver and kidneys, serum biochemical parameters were measured. As shown in Supplementary Table S3, serum ALT, AST, BUN, and Cr levels in the nanoprobe-treated group (34.00 ± 2.24 U/L, 87.00 ± 4.74 U/L, 5.80 ± 0.22 mmol/L, and 42.00 ± 2.24 μmol/L, respectively) were comparable to those in the control group (32.60 ± 2.70 U/L, 85.00 ± 4.74 U/L, 5.64 ± 0.27 mmol/L, and 40.80 ± 2.39 μmol/L, respectively), with no significant differences observed for any parameter (all P > 0.05). These biochemical findings are consistent with the histopathological observations and further confirm that the nanoprobe treatment did not induce detectable hepatic or renal dysfunction. The current proof-of-concept study was conducted exclusively in female mice to minimize inter-individual variability, and that sex-related differences in response to the nanoprobes remain to be investigated in future work.

Taken together, these results establish that the Fe_3_O_4_@Au-HPG-Glc nanoprobe platform not only achieves effective photothermal ablation of osteosarcoma tumors upon NIR irradiation but also does so without inducing overt acute toxicity in major organs. This favorable efficacy-to-safety balance supports the continued development of this image-guided photothermal strategy for targeted cancer therapy.

## 4 Conclusion

In summary, we have successfully developed a novel multifunctional flower-like Fe_3_O_4_@Au-HPG-Glc nanoprobe for image-guided photothermal therapy of osteosarcoma. The nanoprobe integrates the advantages of superparamagnetic Fe_3_O_4_ for T_2_-weighted MRI and gold for NIR photothermal conversion, with an HPG-Glc surface for enhanced stability and tumor targeting. We have shown that the nanoprobes are efficiently taken up by U2OS cells, induce ROS and apoptosis, and exhibit sustained tumor accumulation *in vivo*. Under NIR irradiation, they achieve rapid and effective tumor heating, resulting in significant growth inhibition. The favorable pharmacokinetic profile and lack of acute systemic toxicity further provides a strong foundation for further preclinical development. This platform represents a promising strategy for image-guided, targeted photothermal therapy, offering a potential pathway to overcome the limitations of current osteosarcoma treatments.

## Data Availability

The original contributions presented in the study are included in the article/Supplementary Material, further inquiries can be directed to the corresponding authors.
