# Supplementary material for: A multifunctional Fe3O4@Au-HPG-Glc theranostic platform for MRI/fluorescence-guided photothermal ablation of osteosarcoma
Source: Front Bioeng Biotechnol. 2026 Sep 15;14:1868939. doi: 10.3389/fbioe.2026.1868939 (PMC13619523; doi:10.3389/fbioe.2026.1868939)
Supplement: Supplementary file 1 [file Supplementaryfile1.docx]

**Supporting Information**

**A Multifunctional Fe_3_O_4_@Au-HPG-Glc Theranostic Platform for MRI/Fluorescence-Guided Photothermal Ablation of Osteosarcoma**

Shengren Xiong^1,2^, Xiaomei Zheng^*3^, Zhe Wu^2^, Shenghui Wu^2^, Zhongliang Yao^*4^, Weilin Zheng^*4^

1. Fuzhou Second General Hospital, Fuzhou, Fujian, 350007, China;

2. Department of Orthopaedics, Fujian Medical University Union Hospital, Fujian Medical University, Fuzhou, Fujian, 350001, China;

3. Department of Orthopedics Sports Injury Division, Fujian Medical University Union Hospital, Fujian Medical University, Fuzhou, Fujian, 350001, China;

4. School of Materials and Packaging Engineering, Fujian Polytechnic Normal University, Fuzhou, Fujian, 350300, China.

**Corresponding author:** Weilin Zheng (zhengweilin10@163.com)

**Figure S1** FTIR spectra of the Fe_3_O_4_@Au, Fe_3_O_4_@Au-HPG and Fe_3_O_4_@Au-HPG-Glc nanoprobes.

**Figure S2** Analysis of apoptosis using Annexin V and propidium iodide (PI) double staining.

**Figure S3** Cell viability assessed by CCK-8 assay in osteosarcoma cells after treatment with Fe_3_O_4_@Au-HPG-Glc at indicated concentrations. Data are presented as mean ± SD (n = 5). Statistical significance was determined by one-way ANOVA followed by Dunnett’s post-hoc test. **p < 0.01 vs. Control group (0 nM).

**Figure S4** Cell viability assessed by CCK-8 assay in osteosarcoma cells after treatment with Fe_3_O_4_@Au at indicated concentrations (n=5). Cell viability was normalized to the 5 nM control group. Data are mean ± SD (n = 5). Statistical significance was determined by one-way ANOVA followed by Dunnett’s post-hoc test. *p < 0.05, **p < 0.01 vs. Control group (5 nM).

**Figure S5** Relative cell viability of U2OS cells after various treatments measured by CCK-8 assay. Data are mean ± SD (n = 5). Statistical significance was determined by one-way ANOVA followed by Dunnett’s post-hoc test for comparisons with the control group. ***p < 0.001 vs. control group.

**Figure S6** Representative Live/Dead staining images of U2OS cells after various treatments. Live cells are shown in green (calcein-AM), and dead cells are shown in red (propidium iodide).

**Figure S7** Representative Annexin V/PI flow cytometry scatter plots of U2OS cells after various treatments. (A) Control group; (B) Laser alone group; (C) Fe_3_O_4_@Au-HPG-Glc alone group (NPs); (D) Fe_3_O_4_@Au-HPG-Glc plus laser group (NPs+Laser). Q1: necrotic cells; Q2: late apoptotic cells; Q3: early apoptotic cells; Q4: live cells.

**Figure S8** Representative T_2_-weighted slice used for ROI illustration.

**Figure S9 A.** ROI-based quantitative analysis of tumor T_2_-weighted MRI signal intensity over time, Data are mean ± SD (n = 5). p < 0.001 vs. 0 h (one-way ANOVA with Dunnett’s post-hoc test), **B.** T_2_-weighted MR images from 48 h post-injection.

**Figure S10** Plasma Fe concentration–time profiles by ICP-MS. Data are mean ± SD (n = 3 mice per time point)

**Figure S11** Biodistribution of Fe_3_O_4_@Au-HPG-Glc nanoprobes in major organs (heart, liver, spleen, lung, kidney, and tumor) at 24 h post-injection. Data are expressed as percentage of injected dose per gram of tissue (%ID/g) and presented as mean ± SD (n = 3 mice).

**Figure S12** **A.** Temperature elevation profiles of Fe_3_O_4_@Au-HPG-Glc solutions at different concentrations (0–200 μg/mL) upon 808 nm laser irradiation (1.0 W/cm^2^, 4 min). Data are mean ± SD (n = 3). p < 0.001 vs. 0 μg/mL (one-way ANOVA with Dunnett’s post-hoc test). **B.** Temperature elevation profiles of PBS, Fe_3_O_4_ core, and Fe_3_O_4_@Au-HPG-Glc under 808 nm laser irradiation (1.0 W/cm^2^, 4 min). All samples were tested at 100 μg/mL. Data are mean ± SD (n = 3). p < 0.001 vs. PBS group (one-way ANOVA with Tukey’s post-hoc test).

**Figure S13** **A.** Intratumoral temperature rise curves during NIR laser irradiation (808 nm, 1.0 W/cm^2^, 4 min). Data are presented as mean ± SD (n = 5 mice per group). Significant differences from the PBS group were observed at all time points after 0.5 min for the Fe_3_O_4_@Au-HPG-Glc group and after 1 min for the Fe_3_O_4_ core group (p < 0.01 for all comparisons); **B.** Relative tumor volume changes in each group over 14 days post-treatment. Data are presented as mean ± SD (n = 5 mice per group). Statistical significance was determined by repeated-measures two-way ANOVA followed by Tukey’s post-hoc test. Except for the Fe_3_O_4_ core group at Day 2 (p > 0.05), all comparisons between treatment groups and the PBS group were statistically significant (p < 0.05).

**Table S1** Live/Dead staining results of U2OS cells after various treatments

| **Group** | **Percentage of live cells ( %)** | **Percentage of dead cells (%)** |
| --- | --- | --- |
| Control | 94.7 | 5.3 ± 0.6 |
| Laser | 91.8 | 8.2 ± 0.8 |
| NPs | 89.6 | 10.4 ± 0.9 |
| NPs+Laser | 40.6 | 59.4 ± 2.7 |

Data are presented as mean ± SD (n = 3). p < 0.001 vs. control group.

**Table S2** Annexin V/PI flow cytometry analysis of apoptosis in U2OS cells after various treatments.

| **Group** | **Live cells ( %)** | **Early apoptosis (%)** | **Late apoptosis (%)** | **Necrosis (%)** | **Total apoptosis (%)** |
| --- | --- | --- | --- | --- | --- |
| Control | 95.9 ± 0.9 | 2.82 ± 0.3 | 0.96 ± 0.4 | 0.35 ± 1.1 | 3.78 ± 0.4 |
| Laser | 94.4 ± 0.8 | 4.2 ± 0.3 | 1.2 ± 0.3 | 0.24 ± 0.4 | 5.4 ± 0.5 |
| NPs | 94.0 ± 1.1 | 4.48 ± 0.2 | 1.01 ± 0.3 | 0.14 ± 1.2 | 5.49 ± 0.2 |
| NPs+Laser | 42.0 ± 1.7 | 25.8 ± 1.0 | 25.3 ± 0.7 | 6.95 ± 0.1 | 51.1 ± 1.6 |

Data are presented as mean ± SD (n = 3). p < 0.001 vs. control group.

**Table S3** Serum biochemical parameters in control and Fe_3_O_4_@Au-HPG-Glc-treated mice

| **Group** | **ALT（U/L）** | **ALT-SD** | **AST（U/L）** | **AST-SD** | **BUN（mmol/L）** | **BUN-SD** | **Cr（μmol/L）** | **Cr-SD** |
| --- | --- | --- | --- | --- | --- | --- | --- | --- |
| Control | 32.60 | 2.70 | 85.00 | 4.74 | 5.64 | 0.27 | 40.80 | 2.39 |
| Drug | 34.00 | 2.24 | 87.00 | 4.74 | 5.80 | 0.22 | 42.00 | 2.24 |
